# Matrix gla protein (MGP): an overexpressed and migration-promoting mesenchymal component in glioblastoma

**DOI:** 10.1186/1471-2407-9-302

**Published:** 2009-08-27

**Authors:** Sonja Mertsch, Leon J Schurgers, Kathrin Weber, Werner Paulus, Volker Senner

**Affiliations:** 1 Institute of Neuropathology, University Hospital Münster, Münster, Germany; 2 VitaK Inc. and Cardiovascular Research Institute CARIM, Maastricht, the Netherlands

## Abstract

**Background:**

Recent studies have demonstrated that a molecular subtype of glioblastoma is characterized by overexpression of extracellular matrix (ECM)/mesenchymal components and shorter survival. Specifically, gene expression profiling studies revealed that matrix gla protein (MGP), whose function has traditionally been linked to inhibition of calcification of arteries and cartilage, is overexpressed in glioblastomas and associated with worse outcome.

**Methods:**

In order to analyze the role of MGP in glioblastomas, we performed expression, migration and proliferation studies.

**Results:**

Real-time PCR and ELISA assays confirmed overexpression of MGP in glioblastoma biopsy specimens and cell lines at mRNA and protein levels as compared to normal brain tissue. Immunohistochemistry verified positivity of glial tumor cells for MGP. RNAi-mediated knockdown of MGP in three glioma cell lines (U343MG, U373MG, H4) led to marked reduction of migration, as demonstrated by wound healing and transwell assays, while no effect on proliferation was seen.

**Conclusion:**

Our data suggest that upregulation of MGP (and possibly other ECM-related components as well) results in unfavorable prognosis via increased migration.

## Background

Glioblastomas, which represent astrocytic gliomas of grade IV, are the most common and most malignant intrinsic brain tumors [1]. Despite considerable improvements in surgery, radiation therapy and chemotherapy, the prognosis of patients with glioblastoma remains dismal with median survival of about 15 months. A major reason for this unfavorable outcome is extensive, diffuse invasion of glial tumor cells into surrounding brain tissue, which precludes complete surgical resection and leads to rapid recurrence [2,3]. From the biological point of view, glioma invasion is based on interactions of tumor cells with other neoplastic or non-neoplastic cells and with the cerebral extracellular matrix (ECM).

It has been known for decades that glioblastoma cells *in situ *are able to produce and deposit large amounts of interstitial ECMs such as collagens, fibronectins and laminins, a process that may eventually result in gliosarcomas [4]. Furthermore, once taken into cell culture glioblastoma cells usually produce increased amounts of ECM components, a process which has been designated mesenchymal drift [5,6]. More recent whole genome gene expression analyses have identified a subset of glioblastomas that overexpress transcripts of ECM components, corresponding to a mesenchymal gene expression profile and being associated with worse prognosis [7-9].

Matrix gla protein (MGP) is one of the mesenchymal genes overexpressed in glioblastoma samples [9] as well as in recurrent gliomas undergoing malignant progression [10]. Interestingly, MGP expression in astrocytic tumors appears to be related to grade of malignancy (http://www.ncbi.nlm.nih.gov/geo/, Geo database accession numbers GDS1813 and GDS1962). In addition, *in silico *analyses using the REMBRANDT database (http://rembrandt.nci.nih.gov, accessed 10^th ^march 2009) revealed more than two-fold upregulation of MGP in glioblastomas as compared to non-neoplastic brain tissue, and a correlation (p = 0.0011) between MGP overexpression and worse survival in glioblastomas, suggesting that MGP overexpression is prognostically relevant.

MGP was originally isolated from bone tissue and is also expressed in kidney, lung, heart, cartilage and vascular smooth muscle cells [11]. It is upregulated in a variety of tumors, including ovarian, breast, urogenital and skin cancer [12-16]. MGP is a 79-amino acid ECM protein containing post-translationally modified γ-carboxyglutamic acid residues resulting from vitamin K dependent carboxylation [17,18]. MGP is traditionally considered to be involved in the inhibition of calcification of arteries and cartilage [19], and germline mutations in MGP cause Keutel syndrome, leading to ectopic abnormal calcification and midfacial hypoplasia [20]. Because virtually nothing is known about the mechanisms linking upregulation of MGP and prognosis in gliomas and, more generally, about the function of MGP in tumors, we hypothesized that MGP promotes glioma migration and performed expression and migration analyses.

## Methods

### Cell culture

U373fast and U373slow glioma cell line subpopulations, originating from U373MG cells and selected for fast versus slow migration [21], as well as H4, U343MG, 86HG39 and U373MG glioma cell lines were cultured using standard cell culture conditions in Dulbecco's modified Eagle's minimal essential medium (DMEM) supplemented with 10% fetal calf serum (FCS), 100 U/ml penicillin and 100 μg/ml streptomycin at 37°C in 5% CO_2_.

### Tumor and brain samples

Samples from brain tumor tissues were frozen in liquid nitrogen following resection. The tumors were histologically diagnosed as glioblastoma according to WHO criteria [1]. Frozen section analysis verified that specimens used for RNA and protein extraction were composed of non-necrotic tumor tissue. We used 16 different glioblastoma samples (n = 10 for mRNA and n = 10 for immunohistochemical analysis, four samples were used for both analysis), including samples from 9 females and 7 males. The mean age (± SEM) of the patients at the time of surgery was 52 (± 0.7), with a range of 35–73 years. Two out of these 16 different samples were diagnosed as secondary glioblastoma and 14 were diagnosed as primary glioblastoma. Non-neoplastic, histologically normal cerebral cortices from three adult subjects without neurological symptoms were obtained by autopsy. Approval for using tumor and brain tissues had been obtained by the local ethics commission.

### Immunofluorescence

1 × 10^5 ^cells seeded on glass coverslips and grown under standard conditions for 24 h were rinsed in phosphate-buffered saline (PBS) and fixed using 3.7% formaldehyde in PBS. After permeabilizing with 0.1% Triton X-100 (Sigma, Deisenhofen, Germany) in PBS, cells were blocked with 0.5% BSA/PBS. The mouse monoclonal anti-MGP^3–15 ^antibody (Axxora, Grünberg, Germany) was applied in a 1:200 dilution at 4°C overnight. Alexa Fluor 488 conjugated anti-mouse antibody (A11029, Invitrogen, Karlsruhe, Germany, 1:200 dilution) was used as secondary antibody. Nuclei were stained with Hoechst 33258 and cells were mounted with "Fluoromount" (DAKO, Hamburg, Germany).

### Immunohistochemistry

Immunohistochemical analysis of human glioblastoma tissue was done on acetone-fixed (4°C, 8 min) 5- to 8-μm frozen sections. The mouse monoclonal anti-MGP antibody was applied in a 1:50 dilution at 4°C overnight. For detection we used the biotinylated goat anti-mouse IgG BA2001 (dilution 1:100), a horseradish peroxidase avidin complex and AEC as substrate (secondary antibody and reagents from Vector Laboratories, Burlingame, CA). Slides were counterstained with hematoxylin.

For immunostaining on paraffin embedded tissue [22,23], sections were heated in 0.2% (w/v) citric acid at pH 6.0 in a microwave and "kept warm" for 15 min before washing with TBST (10 mM Tris-HCl, 150 mM NaCl, 0.1% Tween; pH 7.6) and incubation with anti-MGP^3–15 ^(1 mg/ml). The antibody was diluted 1:100 in blocking reagent (TBST/1% BSA). Biotinylated sheep anti-mouse IgG (Amersham Biosciences, Little Chalfont, UK) was used as a secondary antibody, followed by incubation with avidin-linked alkaline phosphatase complex (DAKO); staining was performed with the alkaline phosphatase kit I (Vector Laboratories, Burlingame, CA). All specimens were counterstained using hematoxylin and sections were mounted using imsol-mount.

### ELISA Assay

MGP protein concentrations in cell culture supernatant were quantified using a competitive MGP ELISA kit following manufacturer's instructions (Biomedica, Vienna, Austria). We used cell culture supernatant from cell lines U373fast and U373slow, as well as from the cell lines 86HG39, U373MG and U343MG cultured in DMEM without FCS for 24 h (1 × 10^6 ^cells). Absorption was measured at 450 nm. All samples were analyzed in duplicate.

### Quantitative RT-PCR (qRT-PCR)

Total RNA was isolated from subconfluent cultured cells using the RNeasy Plus Mini kit (Qiagen, Hilden, Germany), and from glioblastoma tissue using TRIzol (Invitrogen, Karlsruhe, Germany). Total RNA (0.5 μg) was transcribed in cDNA with the High Capacity cDNA reverse Transcription Kit (Applied Biosystems, Foster City, CA) in a reaction volume of 20 μl. After cDNA synthesis 1 μl from the reaction volume was utilized for qRT-PCR measurements. Measurements were done using TaqMan GeneExpressions Assay MGP (Hs00179899_m1, Applied Biosystems, Foster City, CA). Relative MGP mRNA levels were calculated and compared between tumor and normal tissue or between siRNA and control transfected cells, respectively. Data were normalized relative to glyceraldehyde-3-phosphate dehydrogenase (GAPDH) using primers GAPDH-for-ACC CAC TCC TCC ACC TTT GAC (bp-position 928–948), GAPDH-rev-CAT ACC AGG AAA TGA GCT TGA CAA (bp-position 1003-980) and the fluorescent labelled probe GAPDH-probe-CTG GCA TTG CCC TCA ACG ACC A (bp-position 956–977) as described previously [21]. The equation fold change = 2^-ΔΔct ^was applied to calculate the relative expression of MGP in siRNA transfected glioma cells versus untransfected glioma cells or tumors versus normal cortex, respectively. All measurements were done in duplicates and the experiments were repeated at least twice.

### RNA interference experiments

U373fast, H4 and U343MG cells were subcultured into 24-well plates till 80% confluence. Transfections were performed using the HiPerfect transfection reagent (Qiagen) and two different small interfering RNAs (siRNAs) directed against MGP (Hs_MGP_4 HP siRNA (SI00645428); target sequence: TAG CAG CAT TAC TGA AAT ACA), Hs_MGP_8 siRNA (SI04357913); target sequence: CTC CCT ACT GCT GCT ACA CAA) and a non silencing negative control (negative control siRNA (1022076); target sequence: AAT TCT CCG AAC GTG TCA CGT) (all siRNAs purchased from Qiagen). Transfections were done according to manufacturer's instructions. Successful knockdown was verified with qRT-PCR and immunofluorescence at 24 h, 48 h and 72 h after transfection. All experiments were done independently for at least three times.

### Wound healing assay

U373fast, H4 and U343MG cells were seeded at a density of 8 × 10^4 ^per well in a 24-well plate. 24 h after siRNA transfection cells were scratched ("wounded") using the back side of a standard 100 μl pipette tip. After being washed three times with PBS scratches including the flanking front lines of cells, were photographed (40-fold magnification). Cells were incubated under standard conditions and migration into the scratched area was photographed 24 h and 48 h after wounding (corresponding to 48 h and 72 h post-transfection). The edges of the wound were marked after scratching (time point 0 h), cells migrating over the edges were counted and the number of cells was determined related to the total area of the scratch. Wound closing was compared between MGP knockdown cells and control transfected cells and evaluated using the analysis FIVE software (Soft Imaging System, Münster, Germany). Differences between two data points were determined by Student's t test where p < 0.05 was considered significant. Experiments were performed independently two times, evaluating 4 – 8 scratches in each experiment.

### Transwell migration assay

Disposable ChemoTx 96-well chemotaxis chambers (#106–8, Neuro Probe, Cabin John, MD) were used for migration studies. Compared to classical Boyden chambers, these filters have hydrophobic masks surrounding each of the 96 filter sites. The masks eliminate the need for a top chamber, because they create surface tension, keeping the cell suspension positioned on the hydrophilic filter site located directly above the bottom wells. Each filter site is 5.7 mm in diameter and pores are 8 μm in diameter. Wells ("lower chambers") were filled with 30 μl of DMEM alone. Polycarbonate filters were positioned on microplates and secured in place with corner pins. 24 h after siRNA mediated knockdown 1 × 10^4 ^U373fast, U343MG or H4 glioma cells in 60 μl DMEM were placed directly onto the filter sites ("upper chambers") and incubated for 24 h at 37°C in 5% CO_2_. Non-migrating cells on the top of the filters were removed by gently wiping the filters with cotton swabs. Migrating cells on the bottom side were fixed for 10 min in 3.7% formaldehyde and stained with Hoechst 33258 for 3 min. Stained cells were then captured using a BX50 microscope (Olympus, Tokyo, Japan) and cells were quantified using morphometry software (analysis FIVE, Soft Imaging System). Differences between two data points were determined by Student's t-test where p < 0.05 was considered significant. Two independent experiments were performed, each of them using 8 fold replicates.

### Proliferation assay

Cellular growth was assessed by (3-(4,5-Dimethylthiazol-2-yl))-2,5-diphenyltetrazolium bromide (MTT) assay. Briefly, U373fast, H4 and U343MG cells were seeded at a density of 1×10^4 ^per well in a 96-well plate 24 h after siRNA transfection. For each time point, an exponential dilution series of cells was used with 1 × 10^5 ^cells as starting dilution. After 4 h, the medium was replaced with 200 μl MTT solution (0.5 mg/ml) and incubated for 3 h at 37°C and 5% CO_2_. MTT solution was discarded and 200 μl of isopropanol was added to dissolve the formazan crystals. Measurement was done at 570 nm using an ELISA reader upon 0 h, 24 h, 48 h and 72 h (corresponding to 24 h, 48 h, 72 h and 96 h after transfection). Experiments were independently performed three times, and 6 – 10 wells were evaluated for each experimental condition. Possible differences were determined by Student's t-test.

## Results

### MGP is upregulated in glioblastoma tissues

**Figure 1:**
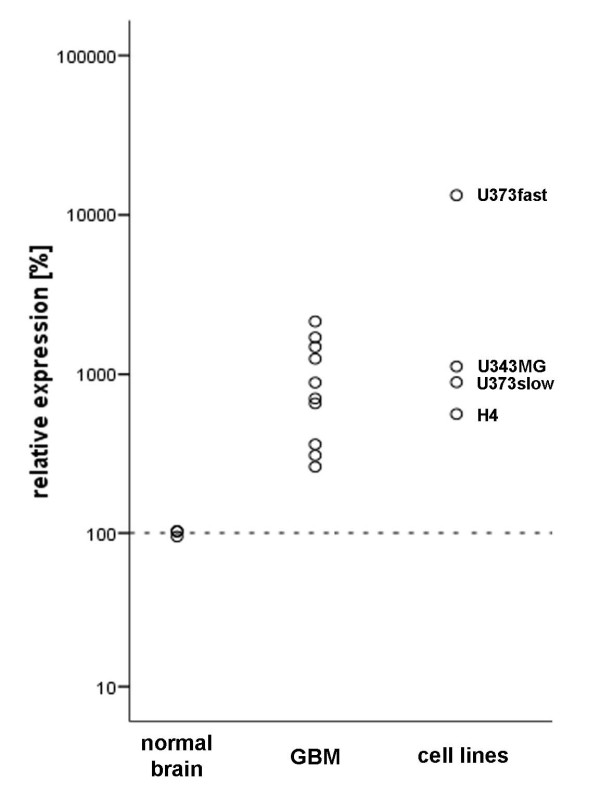
**Expression of MGP mRNA in glioblastoma tissues and cell lines**. Glioblastoma (GBM) biopsy specimens (n = 10) showed increased MGP mRNA levels (quantitative RT-PCR) as compared to normal human brain tissue (n = 3, mean set at 100%). Expression levels of MGP mRNA in glioma cell lines U373fast, U373slow, H4 and U343MG revealed overexpression of MGP mRNA in all cell lines compared to normal brain tissue.

**Figure 2:**
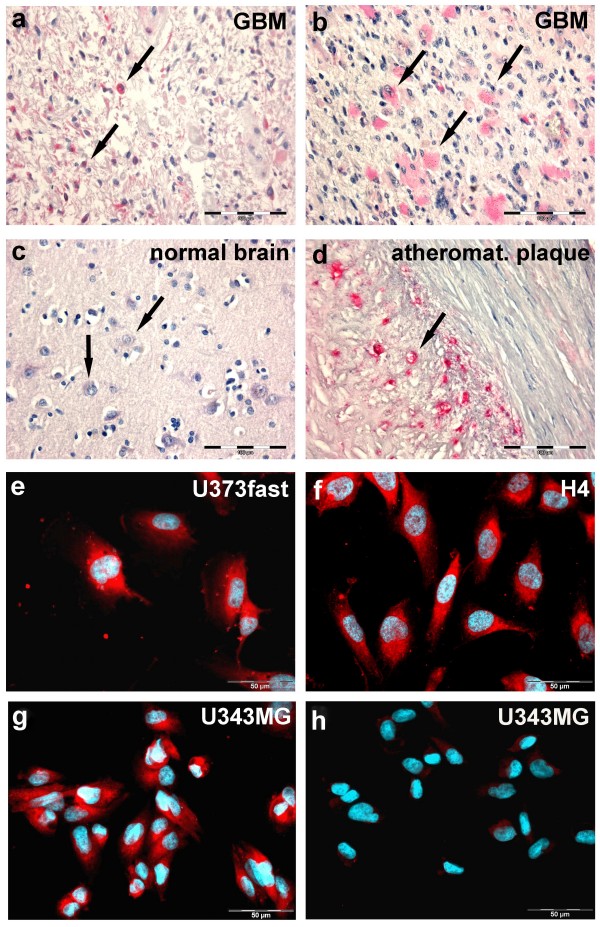
**Cellular localization of MGP protein**. Immunohistochemistry revealed positivity of tumor cells (**a, b, arrow**), while neurons of the normal neocortex were faintly positive (**c, arrow**) and glial cells were negative. An atheromatous plaque, used as positive control, showed distinct staining in the media (**d, arrow**). **e-g**: Immunofluorescence of glioblastoma cell lines U373fast (**e**), H4 (**f**) and U343MG (**g**) showed cytoplasmic positivity. Negative controls using nonspecific IgG remained unlabeled (**h**). Bar = 100 μm (a-d) and 50 μm (e-h).

To determine MGP expression, we first performed quantitative real-time PCR experiments. The amount of MGP mRNA in glioblastoma tissues (n = 10) was 2.6 – 21.4 fold higher compared to normal cortex tissues (n = 3, set as 100%, ± 3.4%) (Figure 1). To verify MGP overexpression on protein levels we performed immunohistochemistry using both frozen and paraffin sections, which showed distinct staining for MGP in tumor cells of all glioblastomas examined (n = 10) (Figure 2a and 2b), while cells of normal autopsy brains were negative or only faintly positive (Figure 2c). An atheromatous plaque, used as positive control, exhibited staining of the media, in part being associated with lipid, which is a normal staining pattern for MGP (Figure 2d). Negative controls did not reveal any staining. MGP mRNA expression did not correlate with age, gender and location of the tumor.

### MGP is upregulated in glioblastoma cell lines

The initial finding of increased MGP expression in glioblastoma tissues compared to normal cortex led us to experiments with glioblastoma cell lines with different migratory phenotype [21]. U373fast cells, characterized by an increased migratory phenotype, demonstrated 22.7-fold higher MGP mRNA expression levels as compared to their slow counterparts (U373slow) and 132.8-fold overexpression compared with normal cortex (Figure 1). Two other glioblastoma cell lines, U343MG and H4, showed 11.2-fold and 5.6-fold higher MGP mRNA expression compared to normal brain tissue (Figure 1). These findings were confirmed at protein levels. ELISA analysis revealed 2.1-fold overexpression of MGP protein in U373fast cells as compared to U373slow cells. We also investigated the glioma cell lines 86HG39, U343MG and non selected U373MG and could verify MGP protein expression in the supernatants using ELISA. The cell line 86HG39 showed a concentration of 0.38 nmol/l MGP protein, U343MG 0.23 nmol/l and U373MG 0.22 nmol/l.

Immunofluorescence showed cytoplasmic staining with accentuation at perinuclear regions in all tested cell lines (Figure 2e–g). Negative controls using isotype matched irrelevant antibodies revealed no staining (Figure 2h).

### MGP is involved in glioma cell migration

**Figure 3:**
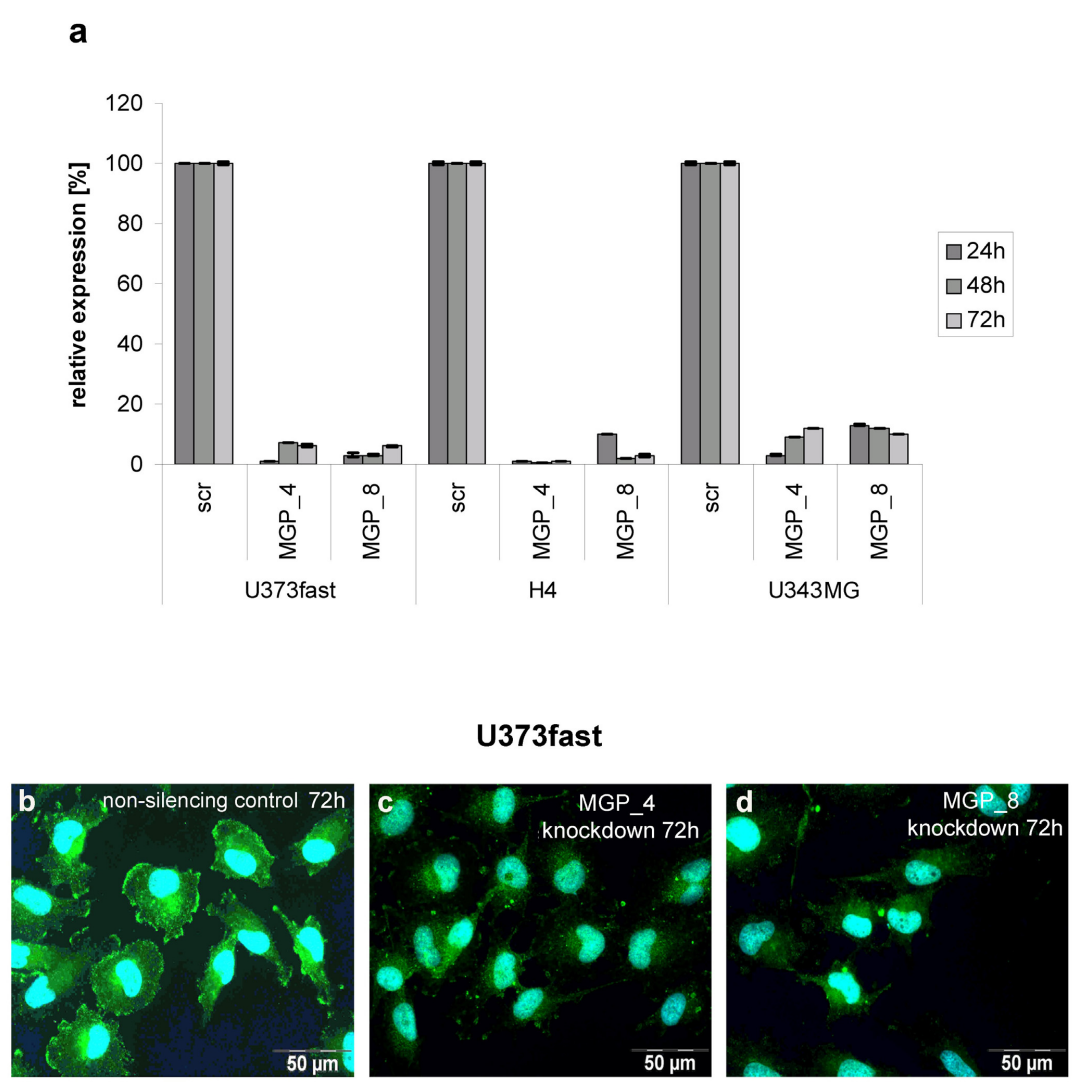
**MGP knockdown using siRNA**. (**a**) Transfection of U373fast, H4 and U343MG cells with two different siRNAs targeting MGP mRNA resulted in decreased MGP expression at mRNA levels at different time points following transfection, as determined by quantitative RT-PCR. MGP mRNA is expressed relative to control transfections using non-silencing siRNA (set at 100%). **b-d**: Knockdown verification on protein levels by immunofluorescence staining revealed decreased fluorescence intensities in U373fast cells for both siRNAs 72 h post-transfection (**c, d**) compared to control transfected cells (**b**) (MGP is marked green in the cytoplasm). Similar results were obtained for H4 and U343MG cells. Nuclei were counterstained with Hoechst 33258 (blue).

To examine the functional role of MGP in glioma cell migration, we first performed siRNA mediated MGP knockdown experiments with U373fast, H4 and U343MG cell lines. MGP knockdown was verified using qRT-PCR, showing residual MGP mRNA levels of 1.1% – 12.9% at 24 h, 0.4% – 11.7% at 48 h, and 0.8% – 11.6% at 72 h after knockdown compared to 100% MGP mRNA expression of control transfected cells (n = 8) (Figure 3a). Accordingly, immunofluorescence revealed reduced fluorescence intensities for both siRNAs, as illustrated for U373fast cells at 72 h post-transfection in Figure 3b–d.

**Figure 4:**
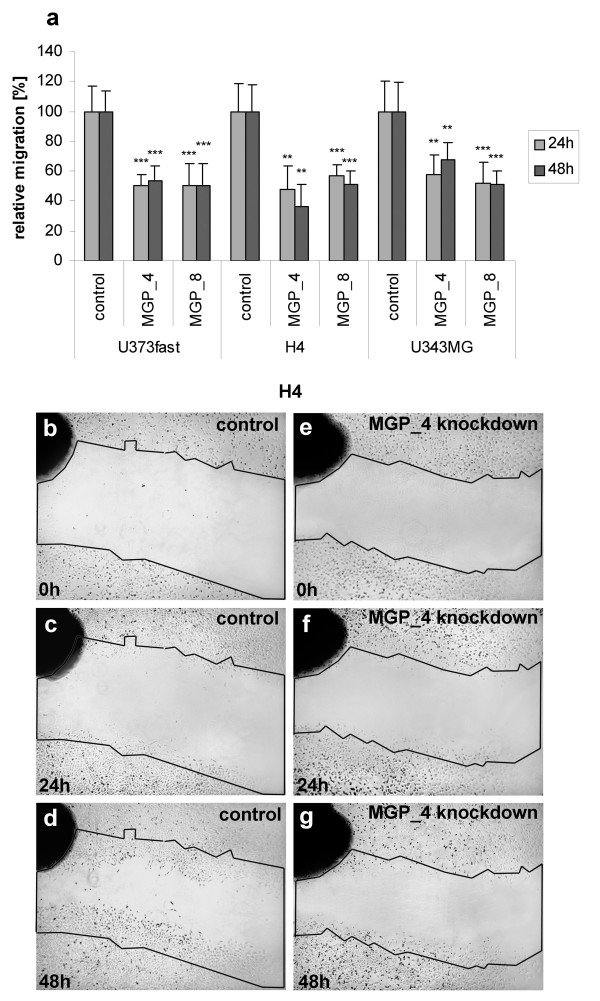
**Migration of glioma cells following MGP knockdown in a wound-healing assay**. (**a**) Quantification of cell migration: U373fast, H4 and U343MG cells with blocked MGP expression exhibited reduced migration as compared to control cells transfected with non-silencing siRNA (set at 100%). All differences between MGP siRNA and cells transfected with control siRNA are significant (p < 0.01 = ** or p < 0.001 = ***). **b-g**: Representative monolayer wound healing assay using H4 cells. For better illustration of differences in migration activity, the initial borders of scratch areas are labeled. H4 cells after MGP knockdown (right panels, **e, f, g**) showed reduced migration into central scratch areas as compared to the control cells, transfected with non-silencing siRNA (left panels, **b, c, d**).

Wound healing assays at 24 h post-wounding (48 h post-transfection) and 48 h post-wounding (72 h post-transfection) showed decreased migration of 50.67% ± 0.07% and 51.74% ± 2.97% for U373fast cells (p = 0.00092 and p = 0.0048 MGP_4, p = 0.00805 and p = 0.0017 MGP_8), 52.67% ± 9.09% and 43.71% ± 14.16% for H4 cells (p = 0.0197 and p = 0.0135 MGP_4, p = 0.00461 and p = 0.00260 MGP_8), and 54.76% ± 5.78% and 59.34% ± 16.38% for U343MG cells compared to control transfected cells (p = 0.0171 and p = 0.0241 MGP_4, p = 0.00974 and p = 0.00714 MGP_8) (Figure 4).

**Figure 5:**
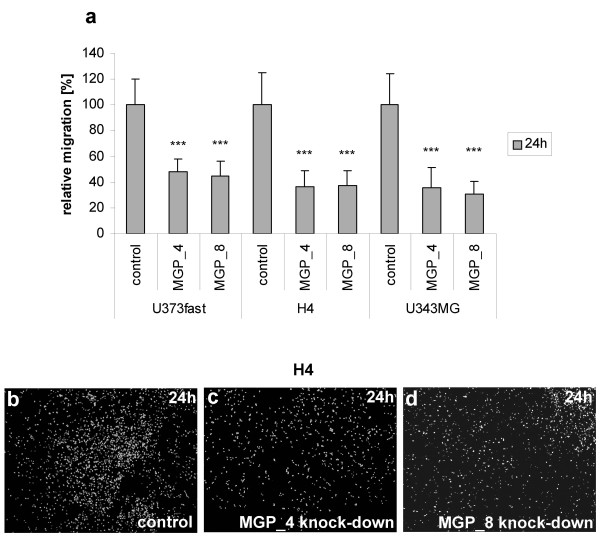
**Migration of glioma cells following MGP knockdown using ChemoTX migration chambers**. (**a**) Quantification of cell migration: U373fast, H4 and U343MG cells with reduced MGP expression exhibited reduced migration as compared to control cells transfected with non-silencing siRNA (set at 100%) 24 h after seeding in the upper chamber (all differences between siRNA and control transfected cells are significant with p < 0.001 = ***). **b-d**: Representative migration chamber assay using H4 cells. Reduced numbers of H4 cells were observed on the lower side of the filter following knockdown with MGP specific siRNA (**c **= siRNA MGP_4, **d **= siRNA MGP_8) as compared cells transfected with non-silencing siRNA (**b**). Nuclei were counterstained with Hoechst 33258.

Transwell migration assays performed with U373fast, H4 and U343MG cells also revealed inhibitory effects on cell migration following MGP knockdown. Compared to control siRNA transfected cells (set at 100%), relative migration at 24 h (48 h after knockdown) following MGP knockdown using two different siRNAs was 47.7% ± 10.3% and 44.5% ± 11.5% in U373fast cells (p = 9.21 × 10^-9 ^and p = 8.33 × 10^-10^), 36.4% ± 12.1% and 37.27% ± 11.2% in H4 cells (p = 5.14 × 10^-10 ^and p = 1.38 × 10^-9^), and 35.8% ± 15.5% and 31% ± 9.3% in U343MG cells (p = 1.48 × 10^-9 ^and p = 3.50 × 10^-11^) (Figure 5).

To exclude that decreased numbers of migrating cells were the consequence of reduced proliferation, we performed proliferation assays. No difference was detected between MGP knockdown cells and control transfected cells (data not shown).

## Discussion

In this study we have demonstrated that MGP expression at mRNA and protein levels is upregulated in glioblastoma tissues and cell lines, confirming previous expression profiling studies using DNA microchip analyses [9,10]. Based on the immunocytochemical demonstration of intracytoplasmic MGP protein in glioblastoma biopsy specimens and glioma cell lines we conclude that production by glial tumor cells rather than mesenchymal elements (e.g. abnormal blood vessels) is responsible for overexpression of this mesenchymal component in gliomas. Furthermore, human glioma cells, selected for high migration capacity (U373fast) showed elevated MGP expression compared to their less migrating counterparts (U373slow). Knockdown of MGP using siRNA in three different glioma cell lines led to decreased cell migration as demonstrated using two different migration assays, whereas proliferation remained unchanged. Collectively, these findings suggest that MGP is involved in glioblastoma cell migration *in vitro*.

While neural MGP expression has previously been found in rat retinal ganglion cells after induction with glia conditioned factors [24], most previous studies have focused on the role of MGP in cartilage, bone and blood vessels, because vascular smooth muscle cells and chondrocytes are the major sites of MGP expression. MGP serves as an inhibitor of cardiovascular calcification as observed in MGP null mice [19,25] and arterial and valvular calcification after warfarin intake [26,27]. In addition, MGP enhances VEGF expression in bovine aortic endothelial cells by increasing the activity of TGF-β1 signalling through activin-like kinase receptor 1 (ALK1) and the SMAD1/5/8 pathway [28]. The functional role of MGP in neoplasia remains unclear, but in line with our findings in gliomas, increased levels of MGP were found in ovarian, breast, urogenital and skin cancer [12-16], although decreased levels of MGP have been found in colon carcinoma [29], and loss of MGP expression in metastatic renal cell carcinoma and prostatic carcinoma compared with primary tumors has been associated with tumor progression and metastasis [15].

The mechanisms linking MGP knockdown and reduced migration of glioblastoma cells remain to be determined, but based on available evidence it is conceivable that interactions with members of the bone morphogenetic protein (BMP) family and the ECM component vitronectin are involved. BMPs belong to the transforming growth factor-beta (TGF-β) superfamily [30]. MGP regulates BMP4 in vascular endothelial cells, and MGP is able to bind BMP2 and promote an association between BMP2 and matrix components; interaction between MGP and BMP2 interferes with binding of BMP2 to its receptor and activation of Smad1 [28,31,32]. Furthermore, BMPs inhibit the tumorigenic potential of human brain tumor-initiating cells [33,34], so that MGP-induced repression of BMP signalling may retain tumor cells in an undifferentiated state with enhanced migration potential [35]. In addition, MGP effects on glioblastoma cell migration might be mediated by binding of MGP to vitronectin [31]. Vitronectin is a multifunctional plasma and ECM protein, which serves roles in cell adhesion, complement activation, coagulation and fibrinolysis [36-39]. MGP might regulate cell migration via vitronectin receptors of the integrin family, which are known to play important roles in glioblastoma migration and invasion [40,41]. Like MGP, vitronectin expression in gliomas is known to increase with tumor grade *in vivo *[40,42]. Of note, the binding of MGP to vitronectin does not interfere with possible interactions of MGP and BMP2, because the binding regions are different [31]. Thus, the migratory promoting effect of MGP on glioblastoma cell lines might be mediated by both BMPs and vitronectin.

Glioma cells in vitro and *in situ *express various ECM components that modulate their microenvironment and promote migration, including collagens, laminins, brevican, tenascin-C and SPARC. These components interact with tumor cells via ECM receptors of the integrin type. Another pair of ECM component/receptor is hyaluronan and CD44 [43]. Several of the components identified in glioma ECMs are permissive substrates enhancing cell migration, but like the interaction of MGP and vitronectin, effects can be modulated by additional ligands or modifications. For example, brevican exposed its promigratory attributes after cleavage by members of the ADAMTS protease family [44] and glioma migration on laminin is stimulated by binding of glycosaminoglycans [45]. These findings suggest that it is stimulation of invasion by MGP and other ECM components, which underlies the unfavorable prognosis of glioblastomas with mesenchymal gene expression profile.

## Conclusion

In conclusion, we have demonstrated that MGP is overexpressed in glioma cells, while siRNA mediated knockdown leads to decreased glioma cell migration. Taking into account previous data on negative prognostic effects of MGP overexpression in glioblastomas, our data suggest that upregulation of MGP in concert with other ECM-related components may result in unfavorable prognosis via increased invasion.

## Competing interests

The authors declare that they have no competing interests.

## Authors' contributions

Sonja Mertsch carried out the molecular studies, participated in functional assays and drafted the manuscript. Leon J. Schurgers carried out the immunohistology staining. Kathrin Weber performed the ELISA. Werner Paulus and Volker Senner conceived of the study, and participated in its design and coordination and helped to draft the manuscript. All authors read and approved the final manuscript.

## Pre-publication history

The pre-publication history for this paper can be accessed here:

http://www.biomedcentral.com/1471-2407/9/302/prepub

## References

[B1] Louis DN Ohgaki H Wiestler OD Cavenee WK Burger PC Jouvet A Scheithauer BW Kleihues P The 2007 WHO classification of tumours of the central nervous system Acta Neuropathol 2007 114 2 97 109 10.1007/s00401-007-0243-4 17618441 PMC1929165

[B2] Claes A Idema AJ Wesseling P Diffuse glioma growth: a guerilla war Acta Neuropathol 2007 114 5 443 458 10.1007/s00401-007-0293-7 17805551 PMC2039798

[B3] Giese A Glioma invasion – pattern of dissemination by mechanisms of invasion and surgical intervention, pattern of gene expression and its regulatory control by tumorsuppressor p53 and proto-oncogene ETS-1 Acta Neurochir Suppl 2003 88 153 162 14531573 10.1007/978-3-7091-6090-9_21

[B4] Kleihues P David N. Louis HO, Otmar D. Wistler, Webster K Glioblastoma WHO Classification of Tumours of the Central Nervous System 2007 4 Lyon: WHO 33 49

[B5] McKeever PE Insights about brain tumors gained through immunohistochemistry and in situ hybridization of nuclear and phenotypic markers J Histochem Cytochem 1998 46 5 585 594 9606106 10.1177/002215549804600504

[B6] Paulus W Huettner C Tonn JC Collagens, integrins and the mesenchymal drift in glioblastomas: a comparison of biopsy specimens, spheroid and early monolayer cultures Int J Cancer 1994 58 6 841 846 10.1002/ijc.2910580616 7523312

[B7] Freije WA Castro-Vargas FE Fang Z Horvath S Cloughesy T Liau LM Mischel PS Nelson SF Gene expression profiling of gliomas strongly predicts survival Cancer Res 2004 64 18 6503 6510 10.1158/0008-5472.CAN-04-0452 15374961

[B8] Phillips HS Kharbanda S Chen R Forrest WF Soriano RH Wu TD Misra A Nigro JM Colman H Soroceanu L Molecular subclasses of high-grade glioma predict prognosis, delineate a pattern of disease progression, and resemble stages in neurogenesis Cancer Cell 2006 9 3 157 173 10.1016/j.ccr.2006.02.019 16530701

[B9] Tso CL Shintaku P Chen J Liu Q Liu J Chen Z Yoshimoto K Mischel PS Cloughesy TF Liau LM Primary glioblastomas express mesenchymal stem-like properties Mol Cancer Res 2006 4 9 607 619 10.1158/1541-7786.MCR-06-0005 16966431

[B10] Boom J van den Wolter M Kuick R Misek DE Youkilis AS Wechsler DS Sommer C Reifenberger G Hanash SM Characterization of gene expression profiles associated with glioma progression using oligonucleotide-based microarray analysis and real-time reverse transcription-polymerase chain reaction Am J Pathol 2003 163 3 1033 1043 12937144 10.1016/S0002-9440(10)63463-3 PMC1868272

[B11] Cancela L Hsieh CL Francke U Price PA Molecular structure, chromosome assignment, and promoter organization of the human matrix Gla protein gene J Biol Chem 1990 265 25 15040 15048 2394711

[B12] Chen L O'Bryan JP Smith HS Liu E Overexpression of matrix Gla protein mRNA in malignant human breast cells: isolation by differential cDNA hybridization Oncogene 1990 5 9 1391 1395 2216462

[B13] Chen Y Miller C Mosher R Zhao X Deeds J Morrissey M Bryant B Yang D Meyer R Cronin F Identification of cervical cancer markers by cDNA and tissue microarrays Cancer Res 2003 63 8 1927 1935 12702585

[B14] Hough CD Cho KR Zonderman AB Schwartz DR Morin PJ Coordinately up-regulated genes in ovarian cancer Cancer Res 2001 61 10 3869 3876 11358798

[B15] Levedakou EN Strohmeyer TG Effert PJ Liu ET Expression of the matrix Gla protein in urogenital malignancies Int J Cancer 1992 52 4 534 537 10.1002/ijc.2910520406 1399132

[B16] Micke P Kappert K Ohshima M Sundquist C Scheidl S Lindahl P Heldin CH Botling J Ponten F Ostman A In situ identification of genes regulated specifically in fibroblasts of human basal cell carcinoma J Invest Dermatol 2007 127 6 1516 1523 10.1038/sj.jid.5700714 17273163

[B17] Price PA Urist MR Otawara Y Matrix Gla protein, a new gamma-carboxyglutamic acid-containing protein which is associated with the organic matrix of bone Biochem Biophys Res Commun 1983 117 3 765 771 10.1016/0006-291X(83)91663-7 6607731

[B18] Price PA Williamson MK Primary structure of bovine matrix Gla protein, a new vitamin K-dependent bone protein J Biol Chem 1985 260 28 14971 14975 3877721

[B19] Luo G Ducy P McKee MD Pinero GJ Loyer E Behringer RR Karsenty G Spontaneous calcification of arteries and cartilage in mice lacking matrix GLA protein Nature 1997 386 6620 78 81 10.1038/386078a0 9052783

[B20] Munroe PB Olgunturk RO Fryns JP Van Maldergem L Ziereisen F Yuksel B Gardiner RM Chung E Mutations in the gene encoding the human matrix Gla protein cause Keutel syndrome Nat Genet 1999 21 1 142 144 10.1038/5102 9916809

[B21] Tatenhorst L Senner V Puttmann S Paulus W Regulators of G-protein signaling 3 and 4 (RGS3, RGS4) are associated with glioma cell motility J Neuropathol Exp Neurol 2004 63 3 210 222 15055445 10.1093/jnen/63.3.210

[B22] Schurgers LJ Teunissen KJ Knapen MH Geusens P Heijde D van der Kwaijtaal M van Diest R Ketteler M Vermeer C Characteristics and performance of an immunosorbent assay for human matrix Gla-protein Clin Chim Acta 2005 351 1–2 131 138 10.1016/j.cccn.2004.08.003 15563881

[B23] Schurgers LJ Teunissen KJ Knapen MH Kwaijtaal M van Diest R Appels A Reutelingsperger CP Cleutjens JP Vermeer C Novel conformation-specific antibodies against matrix gamma-carboxyglutamic acid (Gla) protein: undercarboxylated matrix Gla protein as marker for vascular calcification Arterioscler Thromb Vasc Biol 2005 25 8 1629 1633 10.1161/01.ATV.0000173313.46222.43 15961706

[B24] Goritz C Thiebaut R Tessier LH Nieweg K Moehle C Buard I Dupont JL Schurgers LJ Schmitz G Pfrieger FW Glia-induced neuronal differentiation by transcriptional regulation Glia 2007 55 11 1108 1122 10.1002/glia.20531 17582617

[B25] Murshed M Schinke T McKee MD Karsenty G Extracellular matrix mineralization is regulated locally; different roles of two gla-containing proteins J Cell Biol 2004 165 5 625 630 10.1083/jcb.200402046 15184399 PMC2172384

[B26] Price PA Faus SA Williamson MK Warfarin causes rapid calcification of the elastic lamellae in rat arteries and heart valves Arterioscler Thromb Vasc Biol 1998 18 9 1400 1407 9743228 10.1161/01.atv.18.9.1400

[B27] Schurgers LJ Aebert H Vermeer C Bultmann B Janzen J Oral anticoagulant treatment: friend or foe in cardiovascular disease? Blood 2004 104 10 3231 3232 10.1182/blood-2004-04-1277 15265793

[B28] Bostrom K Zebboudj AF Yao Y Lin TS Torres A Matrix GLA protein stimulates VEGF expression through increased transforming growth factor-beta1 activity in endothelial cells J Biol Chem 2004 279 51 52904 52913 10.1074/jbc.M406868200 15456771

[B29] Fan C Sheu D Fan H Hsu K Allen Chang C Chan E Down-regulation of matrix Gla protein messenger RNA in human colorectal adenocarcinomas Cancer Lett 2001 165 1 63 69 10.1016/S0304-3835(01)00416-5 11248420

[B30] Wozney JM Rosen V Celeste AJ Mitsock LM Whitters MJ Kriz RW Hewick RM Wang EA Novel regulators of bone formation: molecular clones and activities Science 1988 242 4885 1528 1534 10.1126/science.3201241 3201241

[B31] Nishimoto SK Nishimoto M Matrix Gla protein C-terminal region binds to vitronectin. Co-localization suggests binding occurs during tissue development Matrix Biol 2005 24 5 353 361 10.1016/j.matbio.2005.05.004 15982861

[B32] Zebboudj AF Imura M Bostrom K Matrix GLA protein, a regulatory protein for bone morphogenetic protein-2 J Biol Chem 2002 277 6 4388 4394 10.1074/jbc.M109683200 11741887

[B33] Lee J Son MJ Woolard K Donin NM Li A Cheng CH Kotliarova S Kotliarov Y Walling J Ahn S Epigenetic-mediated dysfunction of the bone morphogenetic protein pathway inhibits differentiation of glioblastoma-initiating cells Cancer Cell 2008 13 1 69 80 10.1016/j.ccr.2007.12.005 18167341 PMC2835498

[B34] Piccirillo SG Reynolds BA Zanetti N Lamorte G Binda E Broggi G Brem H Olivi A Dimeco F Vescovi AL Bone morphogenetic proteins inhibit the tumorigenic potential of human brain tumour-initiating cells Nature 2006 444 7120 761 765 10.1038/nature05349 17151667

[B35] Canoll P Goldman JE The interface between glial progenitors and gliomas Acta Neuropathol 2008 116 5 465 77 10.1007/s00401-008-0432-9 18784926 PMC2759726

[B36] Dufourcq P Louis H Moreau C Daret D Boisseau MR Lamaziere JM Bonnet J Vitronectin expression and interaction with receptors in smooth muscle cells from human atheromatous plaque Arterioscler Thromb Vasc Biol 1998 18 2 168 176 9484980 10.1161/01.atv.18.2.168

[B37] Jang YC Tsou R Gibran NS Isik FF Vitronectin deficiency is associated with increased wound fibrinolysis and decreased microvascular angiogenesis in mice Surgery 2000 127 6 696 704 10.1067/msy.2000.105858 10840366

[B38] Podor TJ Peterson CB Lawrence DA Stefansson S Shaughnessy SG Foulon DM Butcher M Weitz JI Type 1 plasminogen activator inhibitor binds to fibrin via vitronectin J Biol Chem 2000 275 26 19788 19794 10.1074/jbc.M908079199 10764803

[B39] Zheng X Saunders TL Camper SA Samuelson LC Ginsburg D Vitronectin is not essential for normal mammalian development and fertility Proc Natl Acad Sci USA 1995 92 26 12426 12430 10.1073/pnas.92.26.12426 8618914 PMC40370

[B40] Gladson CL Cheresh DA Glioblastoma expression of vitronectin and the alpha v beta 3 integrin. Adhesion mechanism for transformed glial cells J Clin Invest 1991 88 6 1924 1932 10.1172/JCI115516 1721625 PMC295768

[B41] Uhm JH Dooley NP Kyritsis AP Rao JS Gladson CL Vitronectin, a glioma-derived extracellular matrix protein, protects tumor cells from apoptotic death Clin Cancer Res 1999 5 6 1587 1594 10389948

[B42] Gladson CL Wilcox JN Sanders L Gillespie GY Cheresh DA Cerebral microenvironment influences expression of the vitronectin gene in astrocytic tumors J Cell Sci 1995 108 Pt 3 947 956 7542670 10.1242/jcs.108.3.947

[B43] Bellail AC Hunter SB Brat DJ Tan C Van Meir EG Microregional extracellular matrix heterogeneity in brain modulates glioma cell invasion Int J Biochem Cell Biol 2004 36 6 1046 1069 10.1016/j.biocel.2004.01.013 15094120

[B44] Hu B Kong LL Matthews RT Viapiano MS The proteoglycan brevican binds to fibronectin after proteolytic cleavage and promotes glioma cell motility J Biol Chem 2008 283 36 24848 24859 10.1074/jbc.M801433200 18611854 PMC3259830

[B45] Aguiar CB Lobao-Soares B Alvarez-Silva M Trentin AG Glycosaminoglycans modulate C6 glioma cell adhesion to extracellular matrix components and alter cell proliferation and cell migration BMC Cell Biol 2005 6 31 10.1186/1471-2121-6-31 16111491 PMC1201133

